# Splicing of Receptor-Like Kinase-Encoding *SNC4* and *CERK1* is Regulated by Two Conserved Splicing Factors that Are Required for Plant Immunity

**DOI:** 10.1093/mp/ssu103

**Published:** 2014-09-29

**Authors:** Zhibin Zhang, Yanan Liu, Pingtao Ding, Yan Li, Qing Kong, Yuelin Zhang

**Affiliations:** ^a^Department of Botany, University of British Columbia, Vancouver, CanadaV6T 1Z4; ^b^National Institute of Biological Sciences, Zhongguancun Life Science Park, Beijing, People’s Republic of China, 102206

**Keywords:** plant immunity, receptor-like kinase, alternative splicing, SUA, RSN2, SNC4, CERK1.

## Abstract

Plant immune receptors belonging to the receptor-like kinase (RLK) family play important roles in the recognition of microbial pathogens and activation of downstream defense responses. The *Arabidopsis* mutant *snc4-1D* contains a gain-of-function mutation in the RLK SNC4 (SUPPRESSOR OF NPR1-1, CONSTITUTIVE4), which leads to constitutive activation of defense responses. Analysis of suppressor mutants of *snc4-1D* identified two conserved splicing factors, SUA (SUPPRESSOR OF ABI3-5) and RSN2 (*R*EQUIRED FOR *SN*C4-1D 2), that are required for the constitutive defense responses in *snc4-1D*. In *sua* and *rsn2* mutants, *SNC4* splicing is altered and the amount of *SNC4* transcripts is reduced. Further analysis showed that SUA and RSN2 are also required for the proper splicing of *CERK1* (*CHITIN ELICITOR RECEPTOR KINASE1*), which encodes another RLK that functions as a receptor for chitin. In *sua* and *rsn2* mutants, induction of reactive oxygen species by chitin is reduced and the non-pathogenic bacteria *Pseudomonas syringae* pv. *tomato* DC3000*hrc*C grows to higher titers than in wild-type plants. Our study suggests that pre-mRNA splicing plays important roles in the regulation of plant immunity mediated by the RLKs SNC4 and CERK1.

## SUMMARY

Two conserved splicing factors, SUA and RSN2, were identified from a suppressor screen of *snc4-1D*. Both are required for alternative splicing of receptor-like kinase (RLK)-encoding *SNC4* and *CERK1* and their functions, suggesting that pre-mRNA splicing plays important roles in the regulation of plant immunity mediated by the RLKs SNC4 and CERK1.

## INTRODUCTION

Plants use a variety of immune receptors to recognize microbial pathogens and initiate downstream signaling cascades and defense responses (Dangl and Jones, 2001). These receptors fall into three main classes. One class encodes intracellular NB-leucine-rich repeats (LRR) resistance (R) proteins which sense effector proteins delivered to plant cells by pathogens to promote pathogenesis (Caplan et al., 2008; Eitas and Dangl, 2010). The other two classes are constituted by proteins belonging to either the transmembrane receptor-like kinase (RLK) or receptor-like protein (RLP) families (Kruijt et al., 2005; Boller and Felix, 2009). RLKs contain an extracellular ligand-binding sequence, a transmembrane region, and a C-terminal intracellular protein kinase domain. RLPs are similar to RLKs except that they lack a cytoplasmic kinase domain. These transmembrane immune receptors detect pathogen effector proteins secreted into the extracellular space surrounding plant cells, as well as conserved molecular features of microbes collectively known as pathogen-associated molecular patterns (PAMPs).

Early evidence suggesting that members of the RLK family are involved in plant defense came from studies of the rice resistance gene *Xa21*. *Xa21* encodes an RLK with extracellular LRRs that recognize *Xanthomonas* species carrying *AvrXa21* (Song et al., 1995). Several RLKs in *Arabidopsis* were later found to be involved in the perception of PAMP signals. The LRR-RLKs FLS2 and EFR function as receptors of bacterial flagellin and translation elongation factor EF-Tu, respectively (Gomez-Gomez and Boller, 2000; Zipfel et al., 2006). BAK1, also an LRR-RLK, functions as the co-receptor of FLS2 and EFR (Chinchilla et al., 2007; Heese et al., 2007). Another RLK CERK1 is involved in the perception of chitin, a component of the fungal cell wall (Miya et al., 2007; Wan et al., 2008). The extracellular region of CERK1 contains three LysM domains, which directly binds chitin (Liu et al., 2012). In addition to its function in immunity against fungi, CERK1 is also involved in the perception of bacterial peptidoglycans (PGNs) (Willmann et al., 2011) and is required for resistance against bacterial infections (Gimenez-Ibanez et al., 2009).

The expression of PAMP receptors was previously shown to be controlled at both transcriptional and posttranscriptional levels. For example, ethylene regulates the transcription of *FLS2* through ethylene-dependent transcription factors EIN3 and EIL1 (Boutrot et al., 2010; Mersmann et al., 2010). In mutants defective in specific components of endoplasmic reticulum (ER) quality control, the accumulation of EFR is dramatically reduced (Li et al., 2009; Lu et al., 2009; Nekrasov et al., 2009; Saijo et al., 2009), suggesting that ER quality control plays an important role in the biogenesis of EFR.

*Arabidopsis SUPPRESSOR OF NPR1-1*, *CONSTITUTIVE4* (*SNC4*) encodes an atypical RLK with two predicted extracellular glycerophosphoryl diester phosphodiesterase domains (Bi et al., 2010). In the *snc4-1D* mutant, a gain-of-function mutation occurred in the intracellular kinase domain, leading to activation of defense responses without the presence of pathogens. The *snc4-1D* mutant plants constitutively express defense marker genes *PATHOGENESIS-RELATED 1* (*PR1*) and *PR2*, and exhibit enhanced resistance to the oomycete pathogen *Hyaloperonospora arabidopsidis* (*H.a.*) Noco2. The constitutive defense responses in *snc4-1D* are partially dependent on NON-RACE-SPECIFIC DISEASE RESISTANCE1 (NDR1) (Century et al., 1997), MAP KINASE SUBSTRATE1 (MKS1) (Andreasson et al., 2005), and the synthesis of jasmonic acid.

To identify additional components required for *snc4-1D*-mediated defense responses, we performed a genetic screen to search for mutations that suppress the dwarf morphology of *snc4-1D*. Here we report the identification and characterization of *rsn1* (*required for snc4-1D*) and *rsn2*. Positional cloning of *RSN1* and *RSN2* revealed that they encode splicing factors required for proper splicing of *SNC4* and *CERK1*, suggesting that alternative splicing plays an important role in PAMP-triggered immunity.

## RESULTS

### Identification and Characterization of *rsn1 snc4-1D* Mutants

**Figure 1:**
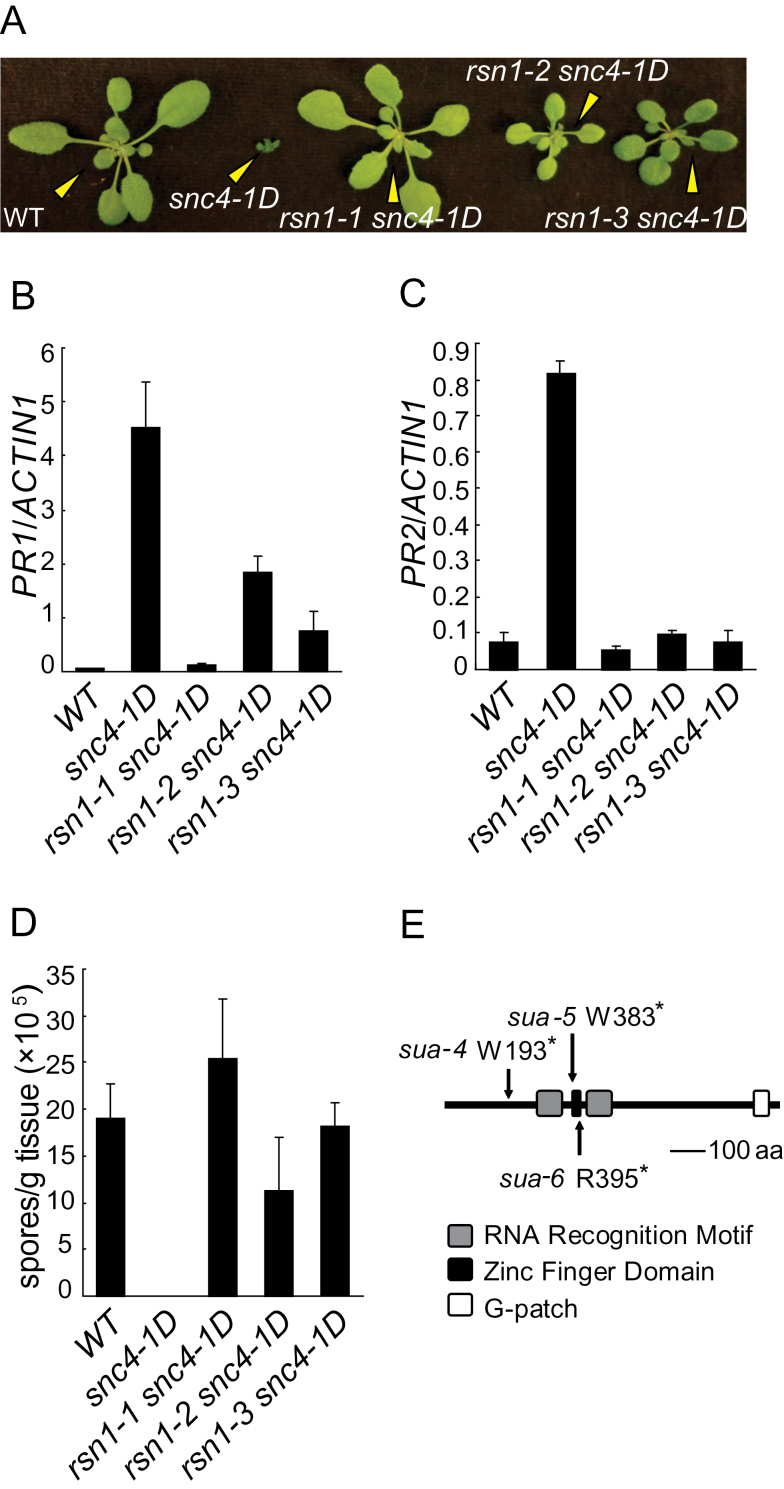
Identification and Characterization of *rsn1* Mutants. **(A)** Morphology of the wild-type (WT), *snc4-1D*, *rsn1-1 snc4-1D*, *rsn1-2 snc4-1D*, and *rsn1-3 snc4-1D* plants. The photograph shows 3-week-old soil-grown plants. **(B, C)** Expression of *PR1* (B) and *PR2* (C) in WT, *snc4-1D*, *rsn1-1 snc4-1D*, *rsn1-2 snc4-1D*, and *rsn1-3 snc4-1D* plants. Values were normalized to the expression of reference gene *ACTIN1*. Error bars represent standard deviations of three repeats. **(D)** Growth of *H.a.* Noco2 in WT, *snc4-1D* and *rsn1-1 snc4-1D*, *rsn1-2 snc4-1D*, and *rsn1-3 snc4-1D* plants. Two-week-old soil-grown seedlings were sprayed with *H.a.* Noco2 spores (5×10^4^ spores ml^–1^). **(E)** The protein structure of RSN1/SUA. Arrows indicate the mutation sites in the protein. aa, amino acid.

From a forward genetic screen to search for mutants that suppress the autoimmune phenotypes of *snc4-1D*, we identified three alleles of *rsn1*. As shown in Figure 1A, *rsn1-1 snc4-1D* has wild-type morphology. *rsn1-2 snc4-1D* and *rsn1-3 snc4-1D* are slightly smaller than wild-type, but much bigger than *snc4-1D*. Analysis of the expression of defense marker genes *PR1* (Figure 1B) and *PR2* (Figure 1C) showed that their expression is much lower in the double mutants than in *snc4-1D*. Furthermore, enhanced resistance to *Hyaloperonospora arabidopsidis* (*H.a.*) Noco2 in *snc4-1D* is abolished in the double mutants (Figure 1D). Taken together, the constitutive defense responses observed in *snc4-1D* are suppressed by the *rsn1* mutants.

### *RSN1* Encodes the Splicing Factor SUA

To map the *rsn1* mutations, *rsn1 snc4-1D* mutants (in the Columbia ecotype background) were crossed with Landsberg to generate a mapping population. Crude mapping of *rsn1-1*, *rsn1-2*, and *rsn1-3* using *snc4-1D*-like F2 plants positioned them to a region between markers T8P19 and MAA21 on chromosome 3. Further mapping showed that they are located between markers F24M12 and T5P19 (Supplemental Figure 1). To identify the *rsn1-2* mutation, a genomic DNA library of *rsn1-2* was generated and sequenced using an Illumina Genome Analyzer. Two mutations that led to changes in amino acid sequence were located between markers F24M12 and T5P19 in *rsn1-2*. One introduced an early stop codon in *At3g54230*. The other mutated Ala-232 in At3g55350 to Thr. Analysis of *rsn1-1* and *rsn1-3* by Sanger sequencing showed that both contain mutations in *At3g54230* (Figure 1E) but not *At3g55350*. These data suggest that *At3g54230* is *RSN1*.

*At3g54230* encodes a splicing factor containing two RNA-recognition motifs (RRMs), a zinc finger domain, and a G-patch motif, which was previously identified as *SUPPRESSOR OF ABI3-5* (*SUA*) (Sugliani et al., 2010). Thus, *rsn1-1*, *rsn1-2*, and *rsn1-3* were renamed as *sua-4*, *sua-5*, and *sua-6*. Among the three alleles, *sua-4* results in the earliest termination of the translation of the protein, giving rise to a short truncated peptide without the RRMs, zinc finger domain, and G-patch motif (Figure 1E). This is consistent with the complete suppression of the *snc4-1D* mutant phenotypes by *sua-4*.

### SUA Is Required for the Splicing of *SNC4*

**Figure 2:**
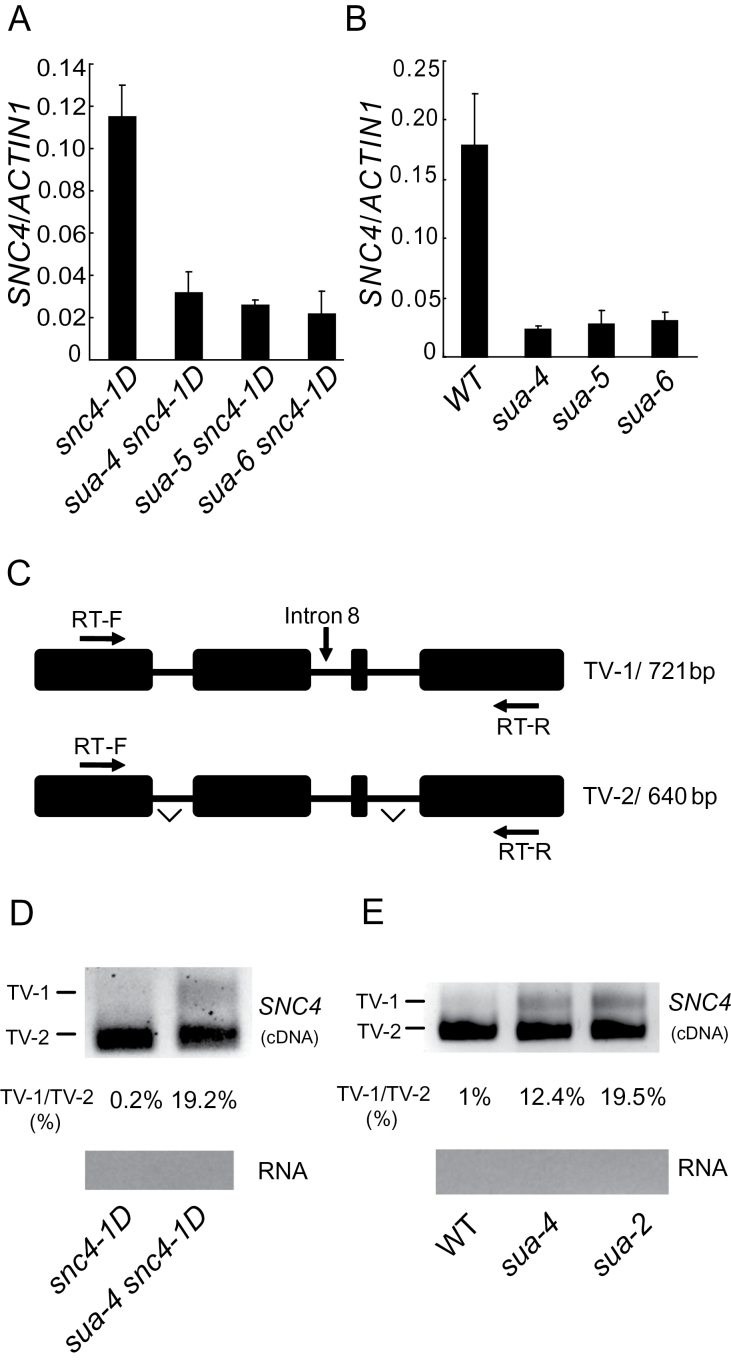
*sua* Mutants Affect the Expression and Splicing Pattern of *SNC4*. **(A, B)** Expression levels of *SNC4* in *snc4-1D* and *sua snc4-1D* double mutants (A) or wild-type and *sua* single mutants (B). Values were normalized to the expression of *ACTIN1*. Error bars represent standard deviations of three measurements. **(C)** Gene structure of the 3’ end of *SNC4*. Exons are indicated with boxes and introns are indicated with lines. Locations of the primers used to amplify the transcript variants are indicated. The lengths of different transcript variants amplified by SNC4-RT-F and SNC4-RT-R are indicated on the right. TV, transcript variant. **(D, E)** Proportions of *SNC4* transcript variants in *snc4-1D* and *sua-4 snc4-1D* (D) or WT, *sua-4*, and *sua-2* (E) plants. RNAs were extracted from 2-week-old seedlings on ½ MS plates and treated with DNase to remove genomic contamination. PCR was performed on cDNA or RNA samples under identical conditions using primer SNC4-RT-F and SNC4-RT-R. cDNA samples used in the PCR reactions were normalized using *ACTIN1*. *sua-2* is a T-DNA insertion mutant of *SUA* (SALK_054379) described previously (Sugliani *et al*., 2010).

SUA is a homolog of the human splicing factor RNA Binding Motif Protein 5 (RBM5). It was shown to be required for alternative splicing of *Arabidopsis ABI3* (Sugliani et al., 2010). To test whether SUA is required for the splicing of *SNC4*, we compared the expression levels of *SNC4* in *snc4-1D*, *sua4 snc4-1D*, *sua5 snc4-1D*, and *sua6 snc4-1D*. As shown in Figure 2A, *SNC4* expression is considerably lower in the *sua snc4-1D* double mutant compared to the *snc4-1D* single mutant. In addition, the expression of *SNC4* in the *sua* single mutants is also lower than in wild-type (Figure 2B).

Next we tested whether splicing of introns in *SNC4* is affected in *sua* mutants. RT–PCR using primers flanking introns revealed that *SNC4* has at least two alternatively spliced transcripts in wild-type: one retaining the eighth intron and one with the intron spliced out (Figure 2C–2E). Since no amplification was detected in PCR reactions using the RNA as templates, the DNA fragments from RT–PCR are not from genomic DNA contamination. In the *sua* mutants, there is a dramatic increase in the intron-retaining transcripts. The identity of the intron-containing transcript was confirmed by sequencing the RT–PCR product.

### SUA Is Required for the Splicing of *CERK1* and PAMP-Triggered Immunity

**Figure 3:**
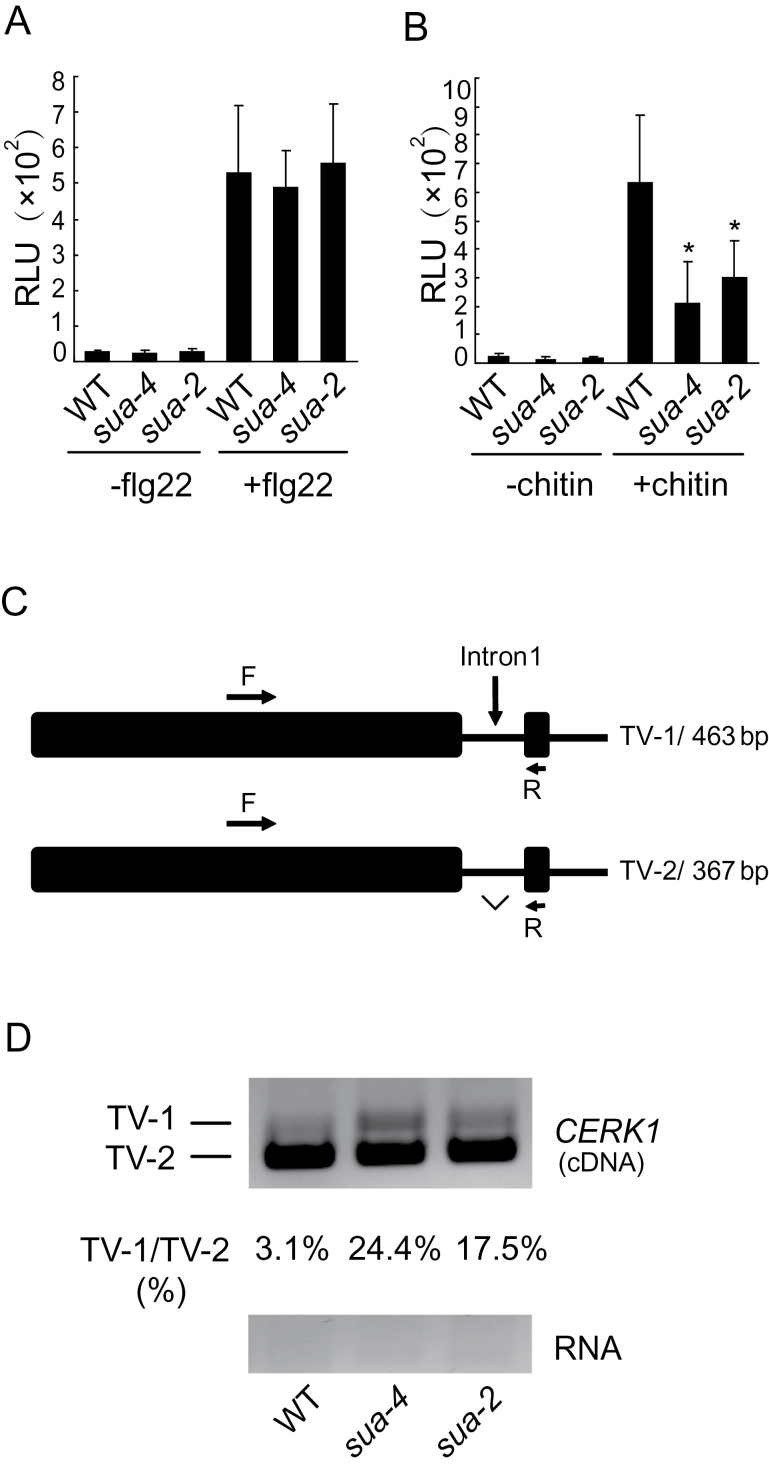
SUA Is Required for the Splicing and Function of *CERK1*. **(A, B)** flg22-induced ROS burst (A) and chitin-induced ROS burst (B) in WT, *sua-4*, and *sua-2* plants. Four-week-old soil-grown plants were used in this assay. Samples were treated with 1 μM flg22 or 200 μg ml^–1^ chitin. Error bars represent standard deviations of 12 repeats. Asterisks above the bars indicate significant difference from WT (*P* < 0.0001). **(C)** Gene structure of the 5’ end of *CERK1*. Exons are indicated with boxes and introns are indicated with lines. Locations of the primers used to amplify the transcript variants are indicated. The lengths of different transcript variants amplified by F and R are indicated on the right. TV, transcript variant. **(D)** Splicing pattern of *CERK1* in WT, *sua-4*, and *sua-2* plants. Total RNA was extracted from 2-week-old plants grown on ½ MS plates and treated with DNase to remove any genomic contaminations. PCR was performed on cDNA or RNA samples using primers CERK1-RT-F (F) and CERK1-SPL-1R (R).

To test whether SUA is required for resistance responses mediated by other RLKs such as FLS2 and CERK1, we assayed flg22 and chitin-induced ROS production in the *sua* mutants. As shown in Figure 3A, induction of ROS production by flg22 is comparable in the wild-type and *sua* mutants. However, chitin-induced ROS production is significantly reduced in the mutant plants (Figure 3B).

To determine whether the splicing of *CERK1* is affected in *sua* mutants, RT–PCR was carried out using primers flanking individual introns. A small amount of the transcript was found to have the first intron retained in the wild-type plants, while a clear increase in the intron-containing transcript was observed in the *sua* mutants (Figure 3C and 3D). Retention of the first intron in the transcript was further confirmed by sequencing the RT–PCR product. These data suggest that SUA is involved in the splicing of *CERK1*.

**Figure 4:**
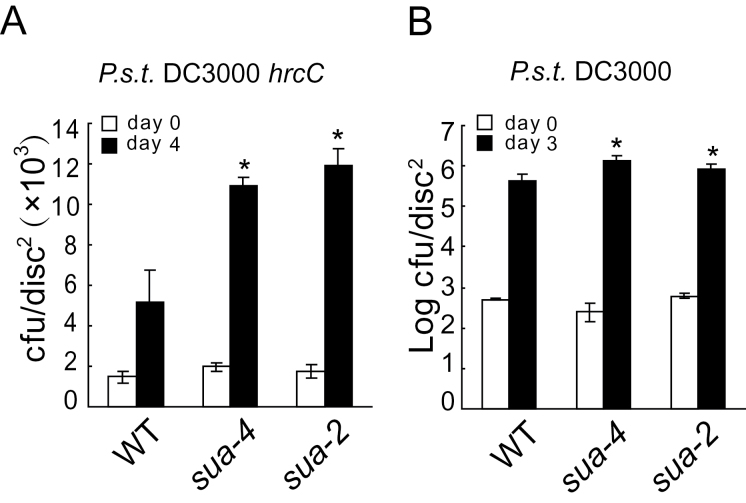
*rsn1* Mutants Compromise PAMP-Triggered Immu nity and Basal Resistance. Growth of *P.s.t.* DC3000*hrc*C **(A)** or *P.s.t.* DC3000 **(B)** on wild-type, *sua-4*, and *sua-2* plants. Four-week-old soil-grown plants were challenged with *P.s.t.* DC3000*hrc*C (OD_600_ = 0.001) or *P.s.t.* DC3000 (OD_600_ = 0.0002). Error bars represent standard deviations of six repeats. Asterisks above the bars indicate significant difference from WT. *P* < 0.001 (A); *P* < 0.05 (B).

To examine whether SUA is required for PAMP-mediated resistance against non-pathogenic bacteria, we challenged *sua* mutants with *Pseudomonas syringae* pv. *tomato* (*P.s.t.*) DC3000*hrc*C. As shown in Figure 4A, *sua* mutants support significantly higher growth of *P.s.t.* DC3000*hrc*C compared to the wild-type plants. When the *sua* plants were challenged with the pathogenic *P.s.t.* DC3000, modestly enhanced susceptibility to the pathogen was also observed in the *sua* mutants (Figure 4B), suggesting that SUA is required for basal defense.

### Identification and Characterization of *rsn2-1 snc4-1D*

**Figure 5:**
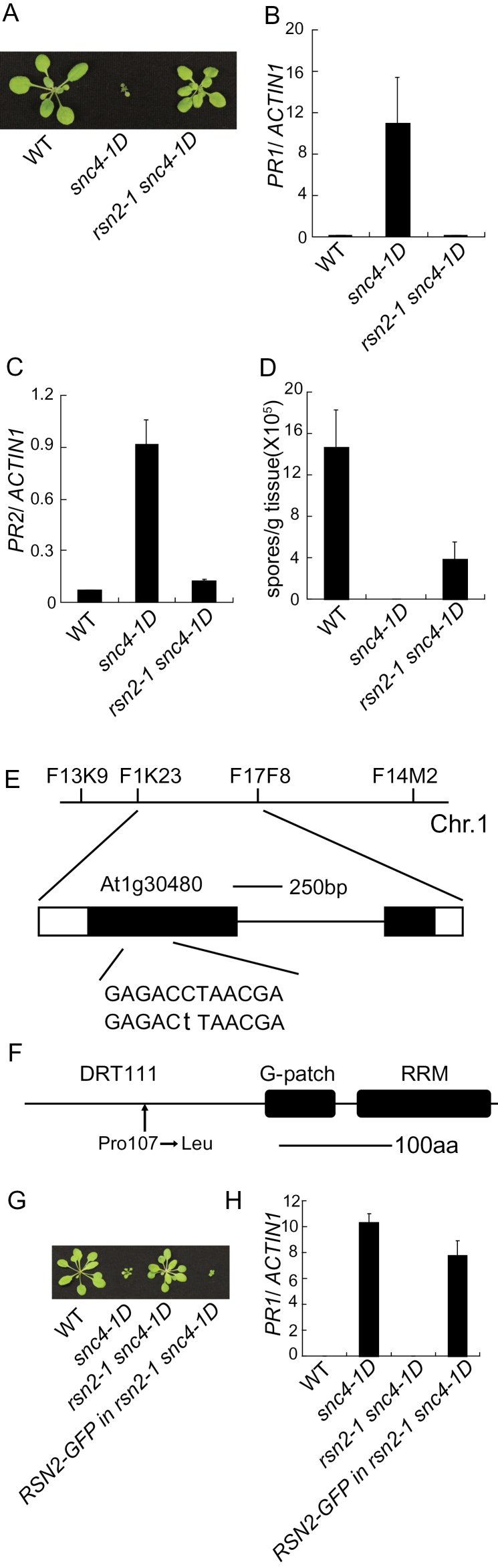
Identification and Characterization of *rsn2-1 snc4-1D*. **(A)** Morphology of wild-type, *snc4-1D*, and *rsn2-1 snc4-1D* plants. Three-week-old soil-grown plants were photographed. **(B)** Growth of *H.a.* Noco2 in wild-type, *snc4-1D*, and *rsn2-1 snc4-1D* plants. Two-week-old soil-grown plants were inoculated with *H.a. Noco2* spores (5×10^4^ spores ml^–1^). **(C, D)** Expression of *PR1* (C) and *PR2* (D) in wild-type, *snc4-1D*, and *rsn2-1 snc4-1D* plants determined by real-time PCR. Total RNA was extracted from 2-week-old seedlings. cDNA was obtained by reverse transcription and subjected to real-time PCR analysis. Values were normalized to the expression of *ACTIN1*. Error bars represent standard deviations of three repeats. **(E)** Map-based cloning of *rsn2-1* and the gene structure of *At1g30480*. The positions of the mapping markers are indicated. Exons are indicated with black boxes. UTRs are indicated with empty boxes and the intron is indicated with a line. **(F)** Protein structure of RSN2. Black boxes indicate conserved domain. RRM stands for RNA-recognition motif. **(G)** Morphological phenotypes of WT, *snc4-1D*, *rsn2-1 snc4-1D*, and *rsn2-1 snc4-1D* with an *RSN2–GFP* transgene. The photograph was taken of 3-week-old soil-grown plants. **(H)** Expression of *PR1* in the indicated genotypes determined by real-time PCR. Values were normalized to expression of *ACTIN1*. Error bars represent standard deviations of three measurements.

Another mutant we identified from the suppressor screen of *snc4-1D* is *rsn2-1*. As shown in Figure 5A, the *rsn2-1 snc4-1D* double mutant exhibits wild-type morphology. In *rsn2-1 snc4-1D*, the expression levels of *PR1* (Figure 5B) and *PR2* (Figure 5C) are much lower than those in *snc4-1D*. Enhanced resistance to *H.a.* Noco2 in *snc4-1D* is also attenuated in *rsn2-1 snc4-1D* (Figure 5D), suggesting that *rsn2-1* mostly suppresses the constitutive defense responses in *snc4-1D*.

### Positional Cloning of *RSN2*

Crude mapping of *rsn2-1* showed that the mutation is located between markers F13K9 and F14M2 on Chromosome 1. Further mapping narrowed the *rsn2-1* mutation to a region of about 0.9Mb between markers F1K23 and F17F8 (Figure 5E). To identify the *rsn2-1* mutation, a genomic DNA library of *rsn2-1* was sequenced by Illumina sequencing. Analysis of the sequence between markers F1K23 and F17F8 identified mutations in three genes. Among them, *At1g30480* encodes a protein previously called DRT111 (DNA-damage-repair/toleration 111), based on its ability to rescue the mutagen-sensitive phenotype of the *Escherichia coli* recG mutant (Pang et al., 1993). It contains a G-patch domain and an RNA-recognition motif (Figure 5F).

Because *At1g30480* encodes a protein with high similarity with the human Splicing Factor 45 (SPF45) (Supplemental Figure 2), we tested whether the mutation in *At1g30480* is responsible for the suppression of *snc4-1D* mutant phenotypes by complementation analysis. A genomic clone expressing At1g30480 with a C-terminal GFP tag under the control of its native promoter was constructed and transformed into *rsn2-1 snc4-1D*. Most of the transgenic lines obtained displayed *snc4-1D*-like dwarf morphology and one representative line is shown in Figure 5G. Quantitative RT–PCR analysis showed that constitutive expression of *PR1* is restored in the transgenic line (Figure 5H). These data suggest that *At1g30480* complements the *rsn2-1* mutation and it is *RSN2*.

### *RSN2* Encodes a Splicing Factor Required for the Alternative Splicing of *SNC4*

**Figure 6:**
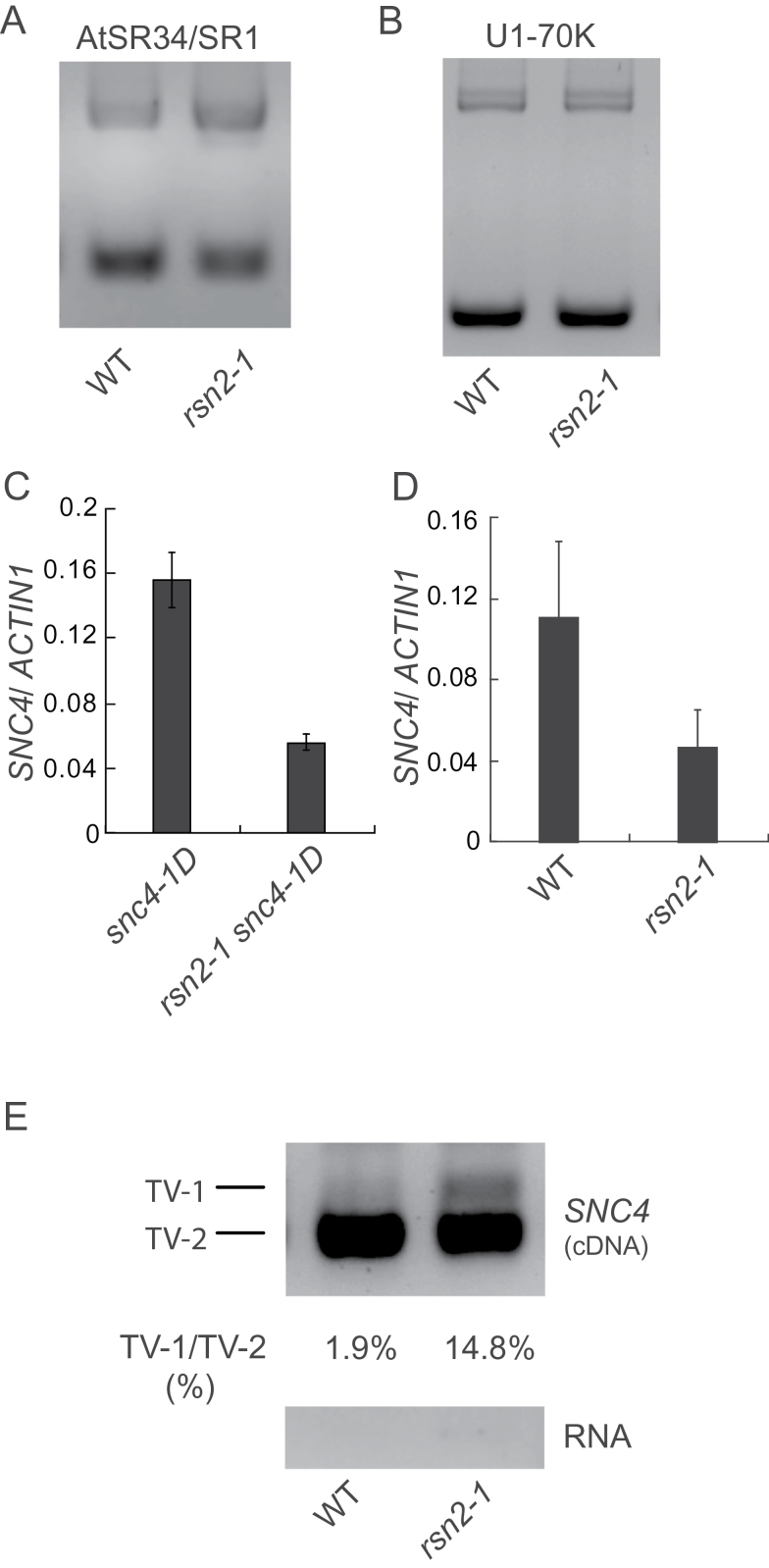
*rsn2-1* Exhibits Altered Splicing Pattern and Expression Reduction of *SNC4*. **(A, B)** Alternative splicing patterns of *AtSR34/SR1* (A) and *U1-70K* (B) in wild-type and *rsn2-1* plants. The primers used were described previously (Golovkin and Reddy, 1996; Savaldi-Goldstein *et al*., 2003). Total RNA was extracted from 2-week-old seedlings grown on ½ MS plates and treated with DNase to remove genomic contamination. cDNA was obtained by reverse transcription. **(C, D)** Expression level of *SNC4* in wild-type and the *rsn2-1* single mutant (C) or *snc4-1D* and the *rsn2-1 snc4-1D* double mutant (D) detected by real-time PCR. Values were normalized to the expression of *ACTIN1*. Error bars represent standard deviations of three measurements. **(E)** Splicing pattern of *SNC4* in WT and *rsn2-1* plants. PCR was performed on cDNA or RNA samples using primers SNC4-RT-F and SNC4-RT-R.

To determine whether RSN2 functions as a general splicing factor, we analyzed the splicing patterns of *AtSR34* and *U1-70K*, two genes previously known to be alternatively spliced (Golovkin and Reddy, 1996; Savaldi-Goldstein et al., 2003). As shown in Figure 6A, the splicing of *AtSR34*, but not *U1-70K*, was affected in *rsn2-1*. This suggests that RSN2 is involved in alternative splicing and that there may be a certain degree of specificity in RSN2-mediated pre-mRNA splicing.

To test whether RSN2 is required for the splicing of *SNC4*, we analyzed the expression level of *SNC4* in *rsn2-1 snc4-1D*. As shown in Figure 6C, *SNC4* expression in *rsn2-1 snc4-1D* is lower than in *snc4-1D* plants. The expression of *SNC4* in the *rsn2-1* single mutant is also lower than in wild-type (Figure 6D). We further tested whether splicing of the eighth intron of *SNC4* is affected in *rsn2-1*. As shown in Figure 6E, the abundance of the intron-containing transcripts is clearly increased in the *rsn2-1*. These data suggest that RSN2 is also required for proper splicing of *SNC4*.

### RSN2 Regulates Alternative Splicing of *CERK1* and Is Required for PAMP-Triggered Immunity

**Figure 7:**
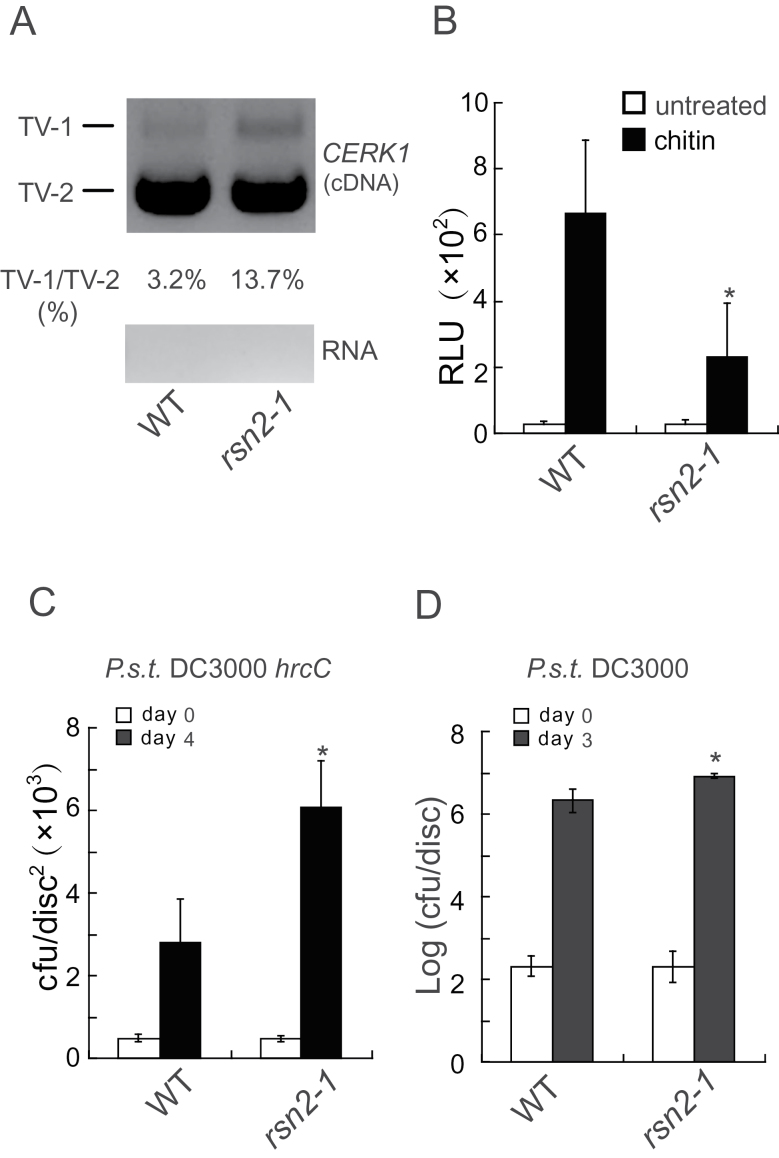
RSN2 Is Required for Alternative Splicing of *CERK1* and PAMP-Triggered Immunity. **(A)** Splicing patterns of *CERK1* in wild-type and *rsn2-1* plants. The primers flanking the first intron were used to amplify a fragment of *CERK1* using cDNA or RNA as templates. **(B)** Chitin-induced ROS burst in wild-type and *rsn2-1* plants. Four-week-old soil-grown plants were used in the assay. Error bars represent standard deviations of 12 repeats. Asterisks indicate significant difference from wild-type (*P* < 0.001). **(C, D)** Growth of *P.s.t.* DC3000*hrc*C (C) or *P.s.t.* DC3000 (D) in wild-type and *rsn2-1* plants. Four-week-old soil-grown plants were challenged with *P.s.t.* DC3000*hrc*C (OD_600_ = 0.001) or *P.s.t.* DC3000 (OD_600_ = 0.0002). Bacterial titers were determined by plating leaf extracts on LB plates. Error bars represent standard deviations of six repeats. Asterisks above the bars indicate significant difference from wild-type. *P* < 0.001 (C); *P* < 0.01 (D).

We then tested whether the splicing pattern of *CERK1* is affected in *rsn2-1* using RT–PCR with primers flanking the first intron. As shown in Figure 7A, the proportion of transcripts retaining the first intron is obviously increased in *rsn2-1*. To determine whether RSN2 is required for CERK1-mediated defense responses, we measure chitin-induced ROS production in *rsn2-1*. As shown in Figure 7B, chitin-induced ROS production is significantly reduced in the mutant plants. In contrast, induction of ROS production by flg22 is comparable between the wild-type and *rsn2-1* (Supplemental Figure 3). To test whether RSN2 is required for PAMP-mediated resistance against non-pathogenic bacteria, we challenged *rsn2-1* mutant plants with *P.s.t.* DC3000*hrc*C. As shown in Figure 7C, significantly higher growth of *P.s.t.* DC3000*hrc*C was observed in *rsn2-1* than in wild-type plants. When *rsn2-1* was challenged with the pathogenic bacteria *P.s.t.* DC3000, a subtle increase in susceptibility to *P.s.t.* DC3000 was also observed. These data suggest that RSN2 is required for proper splicing of *CERK1* and PAMP-triggered immunity.

## DISCUSSION

Pre-mRNA splicing is a critical step in the posttranscriptional regulation of eukaryotic gene expression (Will and Luhrmann, 2011). Multiple mRNAs can be generated from a gene through alternate use of splice sites by the spliceosome, which precisely excise introns and joins exons in pre-mRNA to generate mRNA. Previously, plant NB-LRR resistance genes have been shown to be alternatively spliced (Gassmann et al., 1999; Dinesh-Kumar and Baker, 2000; Halterman et al., 2001; Zhang and Gassmann, 2003; Xu et al., 2011). It was unclear whether alternative splicing also occurs in transcripts of genes encoding other types of immune receptors such as RLKs. In this study, we showed that *SNC4* and *CERK1*, both encoding RLKs, have more than one splicing form, suggesting that alternative splicing also plays an important role in the regulation of these two RLKs.

Our analysis of *snc4-1D* suppressor mutants showed that mutations in two splicing factors, SUA and RSN2, suppress the dwarf morphology and constitutive defense responses in *snc4-1D*. In *sua* and *rsn2* mutant plants, there is increased retention of the eighth intron of *SNC4*. Retention of this intron results in an insertion of 81 nucleotides in the cDNA and 27 amino acids in the protein. In addition, the transcript levels of *SNC4* are also reduced in *sua* and *rsn2* mutant plants. The reduced *SNC4* transcript level is likely caused by degradation of aberrant messages from mis-splicing of *SNC4* and might be the main factor contributing to the suppression of the *snc4-1D* autoimmune phenotypes.

In the *sua* and *rsn2* mutants, the splicing pattern of *CERK1* is also altered and there is increased retention of the first intron. Retention of the first intron introduces an early stop codon, which gives rise to a truncated CERK1 protein with only 219 amino acids. In the *sua* and *rsn2* mutant plants, induction of ROS by chitin is reduced, which could be caused by attenuation of the function of *CERK1*. The non-pathogenic bacteria *P.s.t.* DC3000*hrc*C also grow to higher titers compared to wild-type in the *sua* and *rsn2* mutants. Since *cerk1* mutants exhibit increased susceptibility to *P.s.t.* DC3000*hrc*C (Gimenez-Ibanez et al., 2009), the increased growth of *P.s.t.* DC3000*hrc*C in the *sua* and *rsn2* mutants can be at least partially explained by the mis-splicing and loss of function of *CERK1*.

Spliceosomes are megadalton complexes consisting of five small nuclear ribonucleoprotein particles (snRNPs) and a large number of auxiliary proteins (Will and Luhrmann, 2011). *Arabidopsis* SUA is a homolog of the human splicing factor RBM5, which interacts with the U2 snRNP component U2AF^65^ and functions in splice site selection (Bonnal et al., 2008). RBM5 is involved in the regulation of alternative splicing of apoptotic genes (Bonnal et al., 2008). In addition to its function in the alternative splicing of *SNC4* and *CERK1*, SUA was also shown to be required for the control of alternative splicing of *ABI3* (Sugliani et al., 2010), suggesting that SUA may have functions similar to those of RBM5 and this may play a somewhat general role in the regulation of alternative splicing in *Arabidopsis*.

RSN2 is a homolog of SPF45 in metazoans, which also associates with the U2 snRNP (Will et al., 2002). It was originally identified as a component of the human spliceosome by immuno-precipitation and mass spectrometry (Neubauer et al., 1998). Functional analysis of SPF45 using Hela cell extracts showed that it functions in 3’ splice site selection (Lallena et al., 2002). In *Drosophila*, SPF45 mediates alternative splicing of the *Sex-lethal* pre-mRNA (Chaouki and Salz, 2006). The requirement of RSN2 for correct alternative splicing of *SNC4* and *CERK1* suggests that it may function in pre-mRNA splicing in a manner similar to that of SPF45.

In *Arabidopsis*, both *SUA* and *RSN2* are single copy genes. Among the six *sua* alleles identified, no obvious developmental phenotypes were observed, suggesting that SUA does not function as a general splicing factor and it only regulates the alternative splicing of specific genes. Similarly, the *rsn2-1* mutant does not exhibit apparent pleiotropic defects. The alternative splicing of *SNC4*, *CERK1*, and *AtSR34*, but not *U1-70K*, was found to be altered in *rsn2-1* plants, suggesting that there are also certain degrees of specificity in its splicing function. It remains to be determined whether SUA and RSN2 regulate the splicing of additional RLK-encoding genes and exactly how many genes are regulated by these two splicing factors. Future in-depth genome-wide analysis of genes whose splicing patterns are altered in *sua* and *rsn2* mutants by RNA-sequencing will help us address these questions.

Alternative splicing of genes encoding NB-LRR proteins such as *N*, *RPS4*, and *SNC1* has been shown to be important for their functions in plant immunity (Dinesh-Kumar and Baker, 2000; Zhang and Gassmann, 2003; Xu et al., 2011, 2012). Our study identified two components of the U2 snRNP as critical spicing factors for the alternative splicing of *SNC4* and *CERK1*, which has been shown to impact the functions of the encoded proteins. This suggests that alternative splicing also plays an important role in the regulation of plant immunity mediated by RLKs such as SNC4 and CERK1.

## METHODS

### Plant Growth Conditions and Mutant Screen

All plants used in this study were grown under the condition of 23ºC and 16h light/8h dark cycles unless specifically mentioned. Seeds of *snc4-1D* were obtained by growing the mutant at 28ºC to let it complete its life cycle. *snc4-1D* seeds were mutagenized with EMS as previously described (Bi et al., 2010). About 40 000 M2 plants derived from 2400 M1 lines were grown on soil to identify mutants that lost the dwarf morphology of *snc4-1D*.

### Genome Sequencing and Map-Based Cloning

Genomic libraries used for Illumina sequencing were prepared using NEBNext^®^ DNA Library Prep Reagent Set for Illumina^®^. Briefly, nuclei were extracted from about 2g of plant tissue. Genomic DNA was isolated from the nuclei, broken into small fragments through sonication, and separated by gel electrophoresis. Fragments between 150 and 250bp were recovered using a QIA quick gel extraction kit (Qiagen). End repair was subsequently performed on the recovered DNA followed by adding an ‘A’ base to the 3’ end of the fragments. After that, adaptors were ligated to the DNA fragments and DNA fragments with the adapters were amplified by PCR. The PCR products were recovered and used for sequencing by an Illumina Sequence Analyzer.

To map the *rsn* mutations, the *rsn snc4-1D* mutants in Col background were crossed with L*er* plants to obtain a segregating F2 population. F2 plants with *snc4-1D* morphology were used for crude and fine mapping. Candidate mutations were identified by combining the mapping results and genome sequencing data. Sequences of the markers used for mapping are shown in Supplemental Table 1.

For transgenic complementation of *rsn2-1*, a genomic fragment of *RSN2* was amplified from wild-type genomic DNA using primers RSN2-F and RSN2-R. The PCR fragment was cloned into a modified pCambia1305 vector with an in frame GFP tag at the C-terminus using the *Kpn*I and *Bam*HI restriction sites. The construct was transformed into the Agrobacterium GV3103 and subsequently used to transform *rsn2-1 snc4-1D* plants through floral dip (Clough and Bent, 1998). *rsn2-1 snc4-1D* plants carrying the *RSN2–GFP* transgene were identified by plating the T1 seeds on hygromycin-containing ½ MS medium and transplanted to soil for morphology analysis.

### Gene Expression and Splicing Pattern Analysis

RNA was extracted from 12-day-old plants grown on ½ MS medium using Isol-RNA lysis reagent (5 Prime) and treated with RQ1 RNAase-free DNAase (Promega) to eliminate genomic DNA contamination. cDNA was obtained by reverse transcription and used as templates for subsequent PCR analysis. Real-time PCR was carried out using SYBR Premix Ex Taq II (Takara). The *PR1*, *PR2*, and *ACTIN1* primers for real-time PCR were described previously (Zhang et al., 2003). The primers for analyzing the expression levels of *SNC4* and splicing of the eighth intron of *SNC4* are SNC4-RT-F and SNC4-RT-R. Primers used for analyzing the splicing of first intron of *CERK1* are CERK1-RT-F and CERK1-SPL-1R. The sequences of the primers are shown in Supplemental Table 1.

### Measurement of Oxidative Burst

Individual leaves from 4-week-old soil-grown plants were sliced into six strips (around 1mm × 5mm) and placed into 200 μl ddH_2_O in a 96-well plate. After inoculation for 12h under low light, ddH_2_O were replaced by 200 μl reaction mixture containing 20 μM luminol, 10 μg ml^–1^ peroxidase (sigma), and 1 μM flg22 or 200 μg ml^–1^ chitin. ROS production was determined by recording the luminescence using an Infinite M200 microplate reader.

### Pathogen Infection Assays

For *P.s.t.* DC3000 and *P.s.t.* DC3000*hrc*C infection assays, plants were grown at 23ºC under 12h light/12h dark cycles. Leaves of 4-week-old plants were infiltrated with bacteria suspended in 10mM MgCl_2_. Leaf discs were collected 3 d (*P.s.t.* DC3000) or 4 d (*P.s.t.* DC3000*hrcC*) post inoculation and ground in 10mM MgCl_2_. Bacterial suspensions were diluted and plated on LB medium and colonies were counted 2 d later. For *H.a.* Noco2 infection, 2-week-old soil-grown plants were sprayed with *H.a.* Noco2 (5×10^4^ spores ml^–1^) and kept under the condition of 18ºC, 12h light/12h dark cycles, and >80% humidity for 7 d before growth of *H.a.* Noco2 was quantified as described previously (Bi et al., 2010).

## SUPPLEMENTARY DATA

Supplementary Data are available at *Molecular Plant Online.*

## FUNDING

We are grateful for the financial support to Y.Z. from the Natural Sciences and Engineering Research Council (NSERC) of Canada and the Canada Foundation for Innovation (CFI).

## Supplementary Material

Supplementary Data
