# Supplementary material for: Splicing of Receptor-Like Kinase-Encoding SNC4 and CERK1 is Regulated by Two Conserved Splicing Factors that Are Required for Plant Immunity
Source: Mol Plant. 2014 Sep 29;7(12):1766–75. doi: 10.1093/mp/ssu103 (PMC4261838; doi:10.1093/mp/ssu103)
Supplement: Supplementary Data [file supp_7_12_1766__index.html]

Splicing of Receptor-like kinase-encoding SNC4 and CERK1 is regulated by two conserved splicing factors that are required for plant immunity — Splicing of Receptor-like kinase-encoding SNC4 and CERK1 is regulated by two conserved splicing factors that are required for plant immunity — Splicing of Receptor-Like Kinase-Encoding SNC4 and CERK1 is Regulated by Two Conserved Splicing Factors that Are Required for Plant Immunity — Splicing of Receptor-Like Kinase-Encoding SNC4 and CERK1 is Regulated by Two Conserved Splicing Factors that Are Required for Plant Immunity — Supplementary Data 

# Splicing of Receptor-Like Kinase-Encoding *SNC4* and *CERK1* is Regulated by Two Conserved Splicing Factors that Are Required for Plant Immunity

## Supplementary Data

Data files

**Files in this Data Supplement:**

- Supplementary Data - Supplementary Data
