# Supplementary material for: Splicing of Receptor-Like Kinase-Encoding SNC4 and CERK1 is Regulated by Two Conserved Splicing Factors that Are Required for Plant Immunity
Source: Mol Plant. 2014 Sep 29;7(12):1766–75. doi: 10.1093/mp/ssu103 (PMC4261838; doi:10.1093/mp/ssu103)
Supplement: Supplementary Data [file supp_ssu103_Supplementary_information.pdf]

Supplementary figure legends:

Figure S1. Map-based cloning of *RSN1*.

(A) The positions of the mapping markers. The *rsn1* mutations are flanked between markers F24M12 and T5P19.

(B) Gene structure of *RSN1* and the mutation sites in *RSN1*. Exons are indicated with gray boxes and introns with lines. Arrows indicates the mutation sites of *rsn1* mutants.

Figure S2. Alignment of RSN2 and its homologs from other eukaryotes. Amino acid sequences of hSPF45 from human, SPF45 from *Drosophila*, F58B3.7 from *C.elegans*, OsDRT111 from rice and RSN2 were aligned with Clustal Omega program (http://www.ebi.ac.uk/Tools/msa/clustalo/) and the alignment result was shaded with BOXSHADE program (http://www.ch.embnet.org/software/BOX_form.html).

Figure S3. flg22-induced ROS burst in wild type and *rsn2-1* plants. Four-week-old soil-grown plants were used in this assay.

Supplementary Table S1. Primers used in this study.

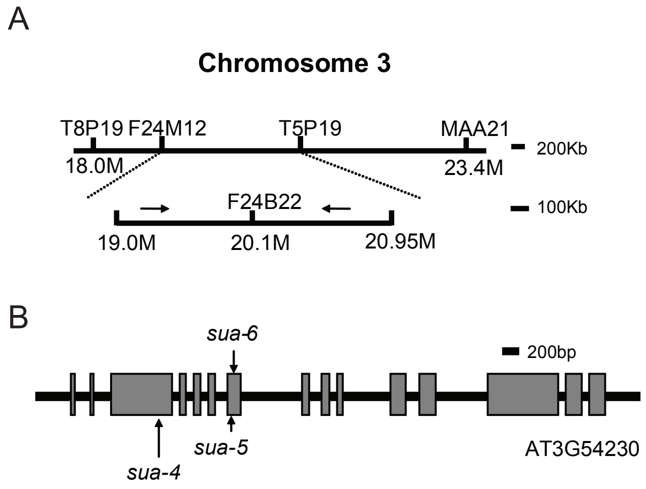

Figure S1. Map-based cloning of *RSN1*.

```
F58B3.7      1 ----MYNDDEDDVPQGPPAAKQAKPMHNIQMAFMQSQLAQRKAALQQQAARQKLVKSSAP
RSN2         1 MLGGLYGDLPPPTDDEKPSGNS-SSVWSSSTKMAPPTLRKPPAF-AP-------------
OSDRT111     1 MLGGLYGDLPPPSSSADDDKPS-AAGWSSAAKMAPPTLRKPPATFAP-------------
hSPF45       1 --MSLYDDLGVETSD-----SK-TEGWSKNFKLIQSQLQVKKAALTQAK-SQRTKQSTVL
SPF45        1 --MDLYDGIDTRARS-----SQ-IDGWSSGIKMLQTQLAVKMAVKK-----------PLM

F58B3.7     57 PPVIDLSTRNRTITTAVTSKSFQPIRANPVSDNISFLPKAATDESVMLFGEEHVKCEYYP
RSN2        46 -PQTILRPLNKPKPIVSAPYKPPPPSNSSQSVLIPANESAPSHQPALVGVTSSVIEEYDP
OSDRT111    47 -PPSVLRN-SRPAPKAPAAQPPPPPTL------PIETTTSTSFQPALVAVQSTVMEEYDP
hSPF45      52 APVIDLKRGGSSDDRQIV-DT-PPHVAAGLKDPVPSGFSAG-------EVLIPLADEYDP
SPF45       42 TPVVNLRSKRLADPEVTC-FA-P--ITTVVSKPLISGKALPSILERINRGDWDVADEYDP

F58B3.7    117 MTPNNYEVVAKEINDRKQREKTAREVAKRLQR-------EHEEEDKKRS-----------
RSN2       105 ARPNDYEEYKREKKRKATEAEMKREMDKRRQEDEERDKREREEREKERERDNSDPSR---
OSDRT111    99 ARPNDYEDYRKDKLKRAKEAEVRKELERRRREEEERERE-REL------REREGRDA---
hSPF45     103 MFPNDYEKVVKRQREE---RQRQRELER---------QKEIEEREKRRK-DR--------
SPF45       98 QRPNEYEKLKEKSNGS---DKNRAGVSD---------REDRDDKEKDRKRGRVGRREFYR

F58B3.7    159 ------------------------------------KGA------AIAPPTMLMEPEPEV
RSN2       162 -------LNISGE----------EA-W--KRRAAMSGGGSGGKGRSSSPPGNVDGFSI--
OSDRT111   149 -------LNISGE----------EA-W--KRRAAMSGSAA---PRPSSSPPHGDGFAI--
hSPF45     142 -------HEASGFARRPDPDSDEDEDYERERRKRSMGGAA------IAPPTSLVEKDKEL
SPF45      146 DEVSAPNLKLSGFGHRQND---DDMYLPSPGLVAKQGGAT------IAPPPSLQEMSI--

F58B3.7    177 IKNTNENQDEKPH----SSFMPPPSFL-------------PAFGKATSRGLGIAANIMKR
RSN2       200 -----------------------------------GKSETSGLGVGAGGQMTAAQRMMAK
OSDRT111   184 -----------------------------------GNSSSSGLGLGAGGQMTAAQRMMAR
hSPF45     189 PRDFPYEEDSRPRSQSSKAAIPPPVYEEQDRPRSPTGPSNSF---LANMGGTVAHKIMQK
SPF45      195 -------------------------------DS--GCEATNT---MPYSASSVAAKIMAK

F58B3.7    220 HGYKEGAGLGKSEQGMSTALSIEKTGVRGGNIVAEAPKAP-T------------------
RSN2       225 MGWKQGQGLGKSEQGITTPLMAKKTDRRAGVIVNASENKSSS------------------
OSDRT111   209 MGWKEGQGLGKQEQGITAPLVAKKTDRRGGVIVDENSS----------------------
hSPF45     246 YGFREGQGLGKHEQGLSTALSVEKTSKRGGKIIVGDATEK--------------------
SPF45      219 YGFKDGQGLGKSEQGMAIALQVEKTSKRGGRIIHEKDVFLPPLALSPPSIGSQIGTSPSH

F58B3.7    261 -----------------FATNSMEAVQNATKILQLWNLTDLSEVSGEEGKKEFADEIKEE
RSN2       267 --------------AEKKVVKSVNINGEPTRVLLLRNMVGPGQV-----DDELEDEVGGE
OSDRT111   247 --------------KQEKKPKSVNFDGPPTRVLLLRNMVGPGEV-----DDELEEEVASE
hSPF45     286 -----------DASKKSDSNPLTEILKCPTKVVLLRNMVGAGEV-----DEDLEVETKEE
SPF45      279 KAMPPPQMVDTAAESGDIGYSITEIMKSPSKVVLLRNMVGPGDV-----DEELEPEVKDE

F58B3.7    304 ME-KCGQVVNVIVHVDE--SQEEDRQVRVFVEFTNNAQAIKAFVMMNGRFFGGRSVSAGF
RSN2       308 CG-KYGTVTRVLIFEITEPNFPVHEAVRIFVQFSRPEETTKALVDLDGRYFGGRTVRATF
OSDRT111   288 CS-KYGTVLRVLIFEITQADFPAEEAVRIFILFERAEEATKAMIDLEGRFFGGRVVRATF
hSPF45     330 CE-KYGKVGKCVIFEIP--GAPDDEAVRIFLEFERVESAIKAVVDLNGRYFGGRVVKACF
SPF45      334 CNTKYGEVNSVIIHESF--GTVPEDAVKIFVEFRRIESAIKAVVDLNGRFFGGRQVRAGF

F58B3.7    361 QNVSDYNNREF-----------
RSN2       367 YDEEKFSKNELAPVPGEIPGY-
OSDRT111   347 FDEERFGKNQLAPMPGEVAGFD
hSPF45     387 YNLDKFRVLDLAEQV-------
SPF45      392 YNYDKFKCFQLH----------
```

Figure S2. Alignment of RSN2 and its homologs from other eukaryotes.

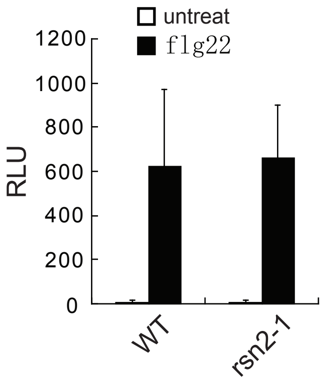

Figure S3. flg22-induced ROS burst in wild type and *rsn2-1* plants.

Table S1. Primers used in this study

Primer	5'-3' sequence	Purpose
SNC4-RT-F	cttcgcagatgaaactgttg	Real-time PCR
SNC4-RT-R	gtggaataagagccttcagc	Real-time PCR
CERK1-RT-F	gcacaatttcagctacag	Real-time PCR
CERK1-RT-R	caacaaagccacaatcactcc	Real-time PCR
CERK1-SPL-1R	ggtgcatttccaccattc	Alternative splicing
AtSR1/SRp34-F	aggagcagaagtcccaaggc	Alternative splicing
AtSR1/SRp34-R	ccttctgaacagaaggtagag	Alternative splicing
U1-70K-F	cccaataatgatccaaatgc	Alternative splicing
U1-70K-R	cttatatgcagctttcatgtc	Alternative splicing
RSN1-F	gagcgggtaccaccaccaccatcatcccgag	Complementation
RSN1-R	cgcggggatccatagccagggatttcacctgc	Complementation
F17F8-F1	ggaagaggattgactcaaagagc	InDel marker
F17F8-R1	gcagcaaacacgtacgtgagc	InDel marker
F1K23-F	gcaaatccataggaagtgagtc	InDel marker
F1K23-R	gatctgtatctgaaacctgggaa	InDel marker
F13K9-F	ttctgctgaaccaaggtgtc	InDel marker
F13K9-R	ataatatgatgcgcgctagg	InDel marker
F14M2-F	cgcatacgtgtcaccgtgag	InDel marker
F14M2-R	tgtccgggactgcctttagc	InDel marker
T8P19-F1	agaacacgcaaaaggacacg	InDel marker
T8P19-R1	gcttagtataactggttttgg	InDel marker
F24M12-F1	tgaagattctacctcgcgtg	InDel marker
F24M12-R1	gttgatctggtgaccaatcc	InDel marker
T5P19-F1	cgtattaaaagtttggagatgttac	InDel marker
T5P19-R1	aatagactgcaaaggtttttgatc	InDel marker
MAA21-F1	gtcacccatatgatgcaaagg	InDel marker
MAA21-R1	gcaattgaagcagactcaacc	InDel marker
F24B22-F1	gtgttgtgtatgtcctgagc	InDel marker
F24B22-R1	cctaaagtacaatgccaagacg	InDel marker
